# Characterization of the Modular Design of the Autolysin/Adhesin Aaa from *Staphylococcus Aureus*

**DOI:** 10.1371/journal.pone.0040353

**Published:** 2012-06-29

**Authors:** Nina Hirschhausen, Tim Schlesier, Georg Peters, Christine Heilmann

**Affiliations:** 1 Institute of Medical Microbiology, University Hospital of Münster, Münster, Germany; 2 Interdisciplinary Center for Clinical Research, University Hospital of Münster, Münster, Germany; University of Osnabrueck, Germany

## Abstract

**Background:**

*Staphylococcus aureus* is a frequent cause of serious and life-threatening infections, such as endocarditis, osteomyelitis, pneumonia, and sepsis. Its adherence to various host structures is crucial for the establishment of diseases. Adherence may be mediated by a variety of adhesins, among them the autolysin/adhesins Atl and Aaa. Aaa is composed of three N-terminal repeated sequences homologous to a lysin motif (LysM) that can confer cell wall attachment and a C-terminally located cysteine, histidine-dependent amidohydrolase/peptidase (CHAP) domain having bacteriolytic activity in many proteins.

**Methodology/Principal Findings:**

Here, we show by surface plasmon resonance that the LysM domain binds to fibrinogen, fibronectin, and vitronectin respresenting a novel adhesive function for this domain. Moreover, we demonstrated that the CHAP domain not only mediates the bacteriolytic activity, but also adherence to fibrinogen, fibronectin, and vitronectin, thus demonstrating for the first time an adhesive function for this domain. Adherence of an *S. aureus aaa* mutant and the complemented *aaa* mutant is slightly decreased and increased, respectively, to vitronectin, but not to fibrinogen and fibronectin, which might at least in part result from an increased expression of *atl* in the *aaa* mutant. Furthermore, an *S. aureus atl* mutant that showed enhanced adherence to fibrinogen, fibronectin, and endothelial cells also demonstrated increased *aaa* expression and production of Aaa. Thus, the redundant functions of Aaa and Atl might at least in part be interchangeable. Lastly, RT-PCR and zymographic analysis revealed that *aaa* is negatively regulated by the global virulence gene regulators *agr* and SarA.

**Conclusions/Significance:**

We identified novel functions for two widely distributed protein domains, LysM and CHAP, i.e. the adherence to the extracellular matrix proteins fibrinogen, fibronectin, and vitronectin. The adhesive properties of Aaa might promote *S. aureus* colonization of host extracellular matrix and tissue, suggesting a role for Aaa in the pathogenesis of *S. aureus* infections.

## Introduction

*Staphylococcus aureus* is one of the most frequently isolated pathogens from community-acquired and nosocomial infections, which range from benign skin infections to serious and life-threatening diseases, such as endocarditis, pneumonia, osteomyelitis, and sepsis [1], [2]. Infections with *S. aureus* may be associated with the use of foreign bodies in modern medicine, such as various catheter systems, prosthetic heart valves, or orthopedic implants [3], [4]. A critical factor in the pathogenesis of these infections is the adherence of *S. aureus* to human tissue or implanted medical devices and the colonization of these surfaces by the formation of multilayered cell clusters that are embedded in an extracellular matrix consisting of polysaccharides, proteins, and DNA called biofilm [5], [6]. The adherence of the staphylococci to biological or artificial surfaces may occur directly or be mediated by plasma and extracellular matrix (ECM) proteins, such as fibrinogen (Fg), fibronectin (Fn), vitronectin (Vn), thrombospondin, or elastin that serve as bridging molecules. Upon adherence, *S. aureus* may be internalized by human host cells thereby evading the host immune system as well as antibiotic treatment by hiding within the host cells, which might explain why some staphylococcal infections are extremely difficult to eradicate [7]. Besides intracellular persistence, the emergence of antibiotic-resistant staphylococci, such as methicillin-resistant *S. aureus* (MRSA) represents an increasing problem in the treatment of *S. aureus* infections. Thus, alternative measures for the treatment and/or prophylaxis of staphylococcal infections are urgently needed.

*S. aureus* expresses a number of genes encoding adhesins that may be classified into different families. One of these families is the MSCRAMM (microbial surface components recognizing adhesive matrix molecules) family, whose members are covalently anchored to the cell wall peptidoglycan and bind to a variety of host factors (reviewed in [8]). Well-studied members of the MSCRAMMs are the Fn-binding proteins FnBPA and FnBPB that bind to Fn, Fg, and elastin and mediate internalization by human host cells [9], [10], [11], [12]. The FnBP-mediated mechanism of *S. aureus* internalization requires Fn as a bridging molecule and the alpha5beta1 integrins as the host cell receptor [13], [14], [15].

Another family of staphylococcal adhesins is represented by the autolysin/adhesins first described by us and others [16]. The autolysin/adhesins have both, enzymatic and adhesive properties and are non-covalently surface-associated via ionic or hydrophobic interactions. In general, autolysins are peptidoglycan hydrolases that are involved in bacterial cell-wall turnover, cell division, cell separation, and antibiotic-induced lysis of bacteria [17]. As the first member of the staphylococcal autolysin/adhesin family, we identified the 148-kDa AtlE from *S. epidermidis* that mediates attachment to polystyrene, adherence to Vn, and biofilm formation [16]. AtlE homologues, such as Aas from *Staphylococcus saprophyticus* [18], AtlC from *Staphylococcus caprae* [19], and Atl from *S. aureus* [20] bind to Fn. Like AtlE, the 137-kDa Atl mediates attachment to polystyrene and biofilm formation [21]. Also, we found that Atl binds to Fg, Vn, and to human EA.hy 926 endothelial cells. Furthermore, both Atl and AtlE mediate internalization by EA.hy 926 cells [20]. Thus, we have identified an alternative staphylococcal internalization mechanism which involves the heat shock cognate protein Hsc70 as the host cell receptor [20].

Further autolysin/adhesins include the 35.8-kDa Aaa from *S. aureus* and the homologous 35-kDa Aae from *S. epidermidis*, which share 65% identical and 76% similar amino acids (aa) [22], [23]. Aaa exhibits bacteriolytic activity against staphylococcal cells, but not against *Micrococcus luteus* cells and mediates adherence to Fg, Fn, and Vn [22]. The Aaa protein contains an N-terminal signal peptide that is followed by three repeated sequences, each of them having high similarity with the lysin motif (LysM), which confers cell wall attachment to various surface-associated proteins (reviewed in [24]). It also contains a C-terminally located cysteine, histidine-dependent amidohydrolase/peptidase (CHAP) domain, which has bacteriolytic activity in many proteins (reviewed in [25], [26]). Aaa has also been characterized by colleagues, who named this protein Sle1 [27]. Sle1 (Aaa) has been shown to be a virulence factor as an *S. aureus sle1* mutant was significantly less virulent than its wild type in a murine acute infection model [27]. Autolysins with adhesive and/or invasive properties have also been reported from other Gram-positive bacteria, such as the peptidoglycan hydrolases P60, Auto, and Ami from *Listeria monocytogenes* [28], [29], [30].

Here, we characterize the functional domain structure of Aaa. We found by biomolecular interaction analysis (BIA) based on surface plasmon resonance (SPR) that both Aaa domains, N-Aaa (LysM) and C-Aaa (CHAP) mediate adherence to the plasma and ECM proteins Fg, Fn, and Vn. Thus, we demonstrated novel adhesive functions for a LysM domain as well as for a CHAP domain. In conclusion, the adhesive properties of Aaa might promote *S. aureus* adherence to host factor-coated material and host tissue thereby potentially contributing to the development of *S. aureus* infections.

## Results

**Figure 1:**
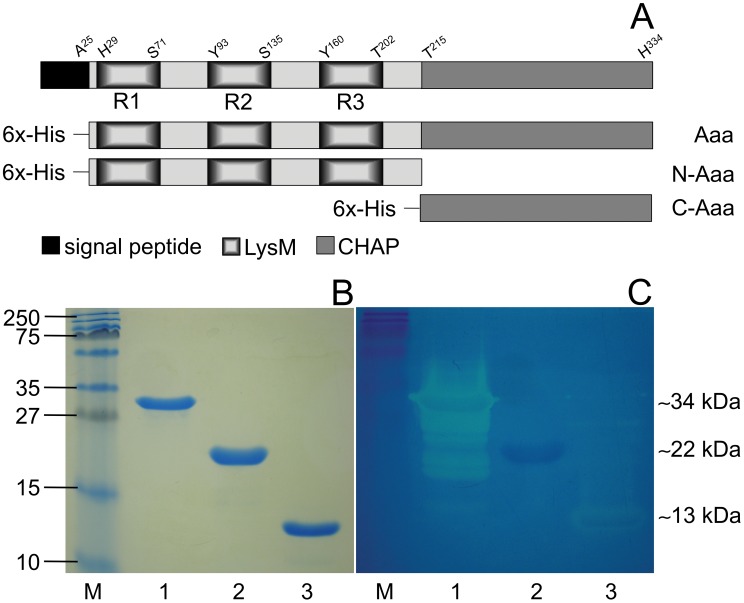
Modular design of Aaa and analysis of recombinant fusion proteins. (A) Schematic model of 6 x His-Aaa, 6 x His-N-Aaa, and 6 x His-C-Aaa. LysM, lysin motif; CHAP, cysteine/histidine-dependent amidohydrolase/peptidase; R1, R2, R3, repeats 1, 2, and 3. (B) SDS-PAGE (15% separation gel) and (C) corresponding zymogram to detect bacteriolytic activity of purified Aaa (lane 1, ∼34 kDa), N-Aaa (lane 2, ∼22 kDa), and C-Aaa (lane 3, ∼13 kDa) against *S. carnosus* cells. Bacteriolytic activity was visible as a clear zone after staining the gel with methylene blue. Aaa and C-Aaa were bacteriolytically active against *S. carnosus*, whereas N-Aaa was detected as a precipitation zone presumably indicating binding to, but not cleavage of peptidoglycan. The sizes of the marker proteins (M; kDa) are indicated on the left.

Previously, we described the identification of the autolysin/adhesin Aaa from *S. aureus* and the cloning and expression of its gene in *E. coli* [22]. To characterize the functional domain structure of Aaa, we expressed and purified the whole Aaa as well as the N-terminal LysM domain (N-Aaa) and the C-terminal CHAP domain (C-Aaa) as His-tagged fusion proteins (Fig. 1A) and investigated their adhesive and bacteriolytic properties. Furthermore, because we recently found that another autolysin/adhesin, Atl from *S. aureus*, binds to and mediates internalization by human host cells [20], we were interested whether Aaa has similar functions.

### Expression, Purification, and Bacteriolytic Activities of 6 x His-Aaa, 6 x His-N-Aaa and 6 x His-C-Aaa Fusion Proteins

For expression of the *aaa* domains in *E. coli*, the PCR-amplified fragments were cloned into the expression vector pQE30. Representative clones producing the N-Aaa and C-Aaa domain contained the plasmids pQaN4074 and pQaC4074, respectively. Subsequently, Aaa, N-Aaa and C-Aaa were purified from the respective *E. coli* clones. SDS-PAGE analysis revealed a ∼34 kDa protein for Aaa (lane 1), a ∼22 kDa protein for N-Aaa (lane 2) and a ∼13 kDa protein for C-Aaa (lane 3) as expected (Fig. 1B). To analyze their bacteriolytic activities, we performed zymographic analysis: SDS-PAGE was performed with a separation gel containing heat-inactivated *S. carnosus* cells as a substrate for lytic enzymes. Aaa and C-Aaa showed a marked zone of bacteriolytic activity at the molecular masses of ∼34 kDa and ∼13 kDa, respectively (Fig. 1C, lanes 1 and 3). Smaller proteins than the ∼34 kDa Aaa protein that showed bacteriolytic activity presumably represented degradation products of Aaa (Fig. 1C, lane 1) and have been observed before with Aaa [22] or other LysM-containing proteins [24]. In contrast, N-Aaa was detected as a precipitation zone at the molecular mass of ∼22 kDa without exhibiting a clear lysis zone presumably indicating binding to, but not bacteriolytic activity, against *S.* *carnosus* cells (Fig. 1C, lane 2).

### Localization of Functional Binding Domains of Aaa and Quantification of Fg-, Fn-, and Vn-binding Activities of Aaa, N-Aaa, and C-Aaa as Determined by Surface Plasmon Resonance (SPR)

**Figure 2:**
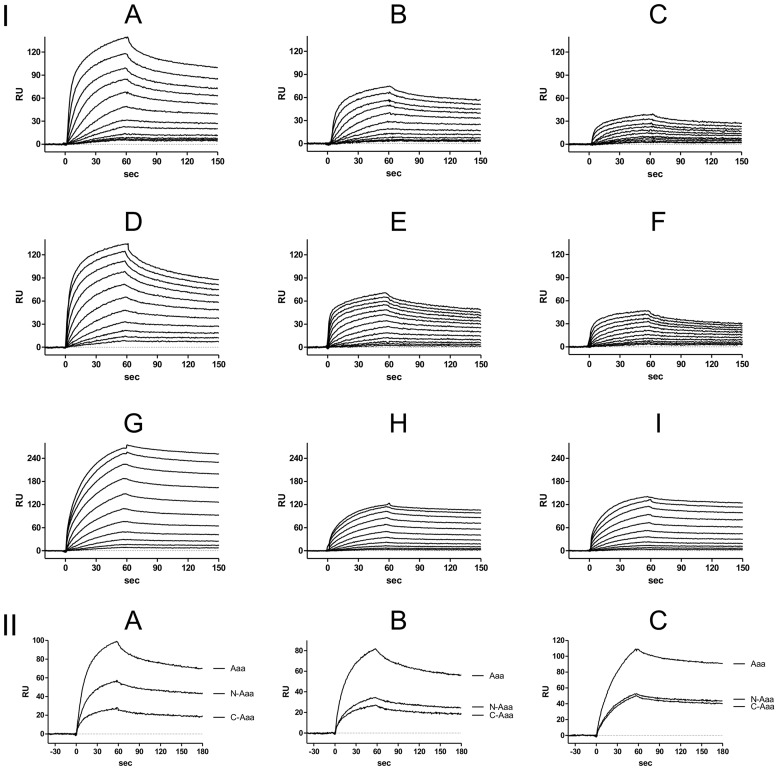
Localization of the functional binding domains within Aaa. Determination of binding of Fg (A, B, C), Fn (D, E, F), and Vn (G, H, I) to the immobilized Aaa (A, D, G), N-Aaa (B, E, H), and C-Aaa (C, F, I) using the BIAcore system. After Aaa, N-Aaa, and C-Aaa were immobilized on the C1 chip surface, Fg, Fn, and Vn were injected over the chip surface at a flow rate of 50 µl/min. I: Binding of Fg, Fn, and Vn was monitored and presented in an overlay-plot of the sensorgrams (a plot of RU [resonance unit] versus time). A, B, C, concentrations of Fg (from bottom to top): 9.16 pM, 18.3 pM, 36.6 pM, 73.2 pM, 146 pM, 293 pM, 586 pM, 1.17 nM, 2.34 nM, 4.69 nM, 9.38 nM, and 18.8 nM. D, E, F, concentrations of Fn (from bottom to top): 293 pM, 586 pM, 1.17 nM, 2.34 nM, 4.69 nM, 9.38 nM, 18.8 nM, 37.5 nM, 75 nM, 150 nM, and 300 nM. G, H, I, concentrations of Vn (from bottom to top): 1.17 nM, 2.34 nM, 4.69 nM, 9.38 nM, 18.8 nM, 37.5 nM, 75 nM, 150 nM, 300 nM, 600 nM, and 1.2 µM. II: Overlay-plot of the sensorgrams (a plot of RU [resonance unit] versus time) of the binding of 4.7 nM Fg to Aaa, N-Aaa, and C-Aaa (A), 18.8 nM Fn to Aaa, N-Aaa, and C-Aaa (B), and 38 nM Vn to Aaa, N-Aaa, and C-Aaa (C) suggesting a 4∶2∶1 stoichiometry for the Fg-binding to Aaa, N-Aaa, and C-Aaa and a 2∶1∶1 stoichiometry for the Vn-binding to Aaa, N-Aaa, and C-Aaa. The stoichiometry for the binding of Fn to Aaa, N-Aaa, and C-Aaa is not completely clear, but appears to be 2∶1∶1.

To localize the functional binding domain(s) within Aaa and to study the adhesive properties of its N-terminal (N-Aaa) and C-terminal (C-Aaa) portions, we performed BIA using SPR [22]. SPR allows the study of interactions between Aaa, N-Aaa, and C-Aaa immobilized on different surfaces of a sensor chip with the ECM proteins Fg, Fn, or Vn as analytes in solution. After Aaa, N-Aaa, and C-Aaa were immobilized on the C1 chip, various concentrations of Fg (from 9.16 pM to 18.8 nM), Fn (from 293 pM to 300 nM) or Vn (from 1.17 nM to 1.2 µM) were sequentially injected over the immobilized proteins for kinetic studies and the sensorgrams of the binding of the ECM proteins to Aaa, N-Aaa, and C-Aaa were monitored as shown in Fig. 2. The interactions of Aaa, N-Aaa, and C-Aaa with Fg, Fn, and Vn were dose-dependent and became biphasic at higher concentrations, indicating a secondary binding event (Fig. 2 I A–I). From the quantitative data of the kinetics of the initial phases of these interactions, dissociation constants (K_D_) were determined to be 0.76 nM, 0.41 nM, and 0.74 nM for the interactions of Aaa, N-Aaa, and C-Aaa, respectively, with Fg, 6.0 nM, 5.1 nM, and 6.8 nM for the interactions of Aaa, N-Aaa, and C-Aaa, respectively, with Fn, and 18 nM, 18 nM, and 20 nM for the interactions of Aaa, N-Aaa, and C-Aaa, respectively, with Vn.

### Complementation of *S. aureus* 4074*aaa* and Characterization of the Adhesive and Invasive Properties of *S. aureus* 4074, 4074*aaa*, and 4074*aaa* (pRCK*aaa*)

**Figure 3:**
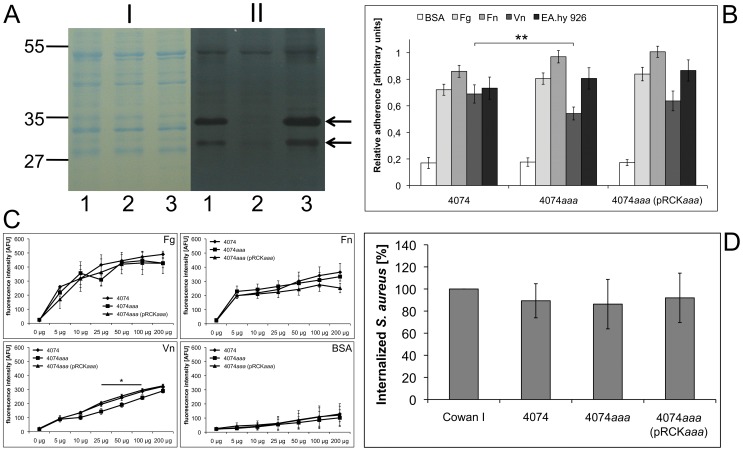
Functional characterization of the adhesive and invasive properties of *S. aureus* 4074, 4074*aaa*, and 4074*aaa*(pRCK*aaa*). A: Complementation of the 4074*aaa* mutant. SDS-PAGE (I; 10% separation gel) and zymographic analysis (II) of surface-associated proteins (10 µl) of *S. aureus* 4074 (lane 1), the 4074*aaa* mutant (lane 2), and the complemented mutant *S. aureus* 4074*aaa* (pRCK*aaa*) (lane 3). The ∼34 kDa band with lytic activity corresponding to Aaa and its ∼28 kDa putative degradation product were missing from the 4074*aaa* mutant (lane 2) and present in the wild type (lane 1) and the complemented mutant (lane 3). The arrows indicate Aaa-associated bacteriolytic activity. The sizes of marker proteins (kDa) are indicated on the left. B: Adherence of *S. aureus* 4074, 4074*aaa*, and 4074*aaa* (pRCK*aaa*) to immobilized Fg, Fn, Vn, and EA.hy 26 cells was assessed by ELISA adherence assays. Binding of the 4074*aaa* mutant to Vn was significantly decreased in comparison to the wild type. In contrast, binding of the 4074*aaa* mutant to Fg, Fn, and EA.hy 926 cells was not altered. As a negative control, binding to BSA was assessed. Results are shown as the mean of three independent experiments. Statistical significance is marked by asterisks. C: As a more sensitive method, flow cytometry was applied to determine the binding of FITC-labeled, soluble plasma proteins to *S. aureus* 4074, 4074*aaa*, and 4074*aaa* (pRCK*aaa*). Again, significantly reduced binding of the 4074*aaa* mutant was only observed to Vn, whereas its binding to Fg and Fn was unchanged. As a negative control, binding to BSA was assessed. The results are shown as the mean of three independent experiments and statistical significance is marked by asterisks. D: The internalization of strains 4074, 4074*aaa*, and 4074*aaa* (pRCK*aaa*) by EA.hy 926 cells was assessed by flow cytometry and computed in relation to the highly invasive strain *S. aureus* Cowan 1, which was set to 100% internalization. The internalization of the 4074*aaa* mutant was in the same range as that of the 4074 wild type and the complemented mutant indicating that Aaa does not play a role in internalization. Results are shown as the mean of three independent experiments.

Because we found that purified recombinant Aaa binds to plasma and ECM proteins as well as to EA.hy 926 cells (data not shown), we sought to characterize the role of Aaa as an adhesin and internalin in the context of the bacterial cell. For this, we cloned the *aaa* gene in the vector pRB473 and introduced the resultant plasmid pRCK*aaa* into *S. aureus* 4074*aaa*. To verify the production and bacteriolytic activity of Aaa encoded on the plasmid pRCK*aaa*, surface-associated proteins of *S. aureus* 4074, the 4074*aaa* mutant, and the complemented mutant 4074*aaa* (pRCK*aaa*) were studied by SDS-PAGE (10% separation gel; Fig. 3A I) and zymographic analysis (Fig. 3A II). The 4074*aaa* mutant (lane 2) clearly lacked the distinct ∼34 kDa band corresponding to Aaa and its putative ∼28 kDa degradation product, which are both present in the 4074 wild type (lane 1) and the complemented mutant 4074*aaa* (pRCK*aaa*) (lane 3). Thus, introduction of the plasmid pRCK*aaa* completely restored the production of Aaa and its bacteriolytic activity.

In order to investigate the adhesive properties of *S. aureus* 4074, the 4074*aaa* mutant, and the complemented mutant 4074*aaa* (pRCK*aaa*), we performed ELISA (Fig. 3B) and flow-cytometric (Fig. 3C) adherence assays. In the ELISA adherence assay, the attachment of the 4074*aaa* mutant to surface-immobilized Vn was significantly reduced in comparison to its wild type (Fig. 3B). In contrast, the binding of the 4074*aaa* mutant to surface-adsorbed Fg, Fn, and endothelial cells was comparable with that of the wild type and the complemented mutant (Fig. 3B). Additionally, we performed flow-cytometric adherence assays that analyzes the binding of soluble, FITC-labeled plasma proteins to the *S. aureus* strains mentioned above. Again, we observed a decreased adherence of the 4074*aaa* mutant only to Vn; whereas the adherence of the 4074*aaa* mutant to Fg and Fn was not significantly altered compared to the wild type and the complemented mutant (Fig. 3C). Binding of all strains to BSA was negligible (Fig. 3B, C).

Recently, we found that the *S. aureus* Atl not only mediates binding to Fn and EA.hy 926 cells, but also mediates the uptake of *S. aureus* by endothelial cells [20]. Therefore, we sought to analyze, whether Aaa analogously might be able to mediate *S. aureus* internalization. To address this question, we compared the uptake of *S. aureus* 4074, the 4074*aaa* mutant and the complemented 4074*aaa* mutant by EA.hy 926 cells using a flow-cytometric internalization assay. The internalization of all three 4074 strains by EA.hy 926 cells revealed no significant differences and was very similar to that of Cowan 1 (Fig. 3D).

### Expression of *aaa* is Increased in the *S. aureus* SA113*atl* Mutant and Vice Versa

*S. aureus* expresses a variety of surface-associated and covalently-anchored surface proteins with redundant adhesive functions, which might explain the mostly unchanged adhesive phenotype of the 4074*aaa* mutant compared to its wild type. Here, it might even be explained by an increased expression of Atl that also binds to plasma and ECM proteins as well as endothelial cells [20] thereby potentially compensating for the loss of Aaa. Earlier observations by us and others suggested that the production of Atl might be increased in the 4074*aaa* mutant and vice versa, the production of Aaa might be increased in the SA113*atl* mutant (unpublished and [21]). Thus, we speculated that the bacteria compensate for the loss of one autolysin (Aaa or Atl) by the upregulation of the other autolysin (Atl or Aaa) by so far unknown mechanisms.

**Figure 4:**
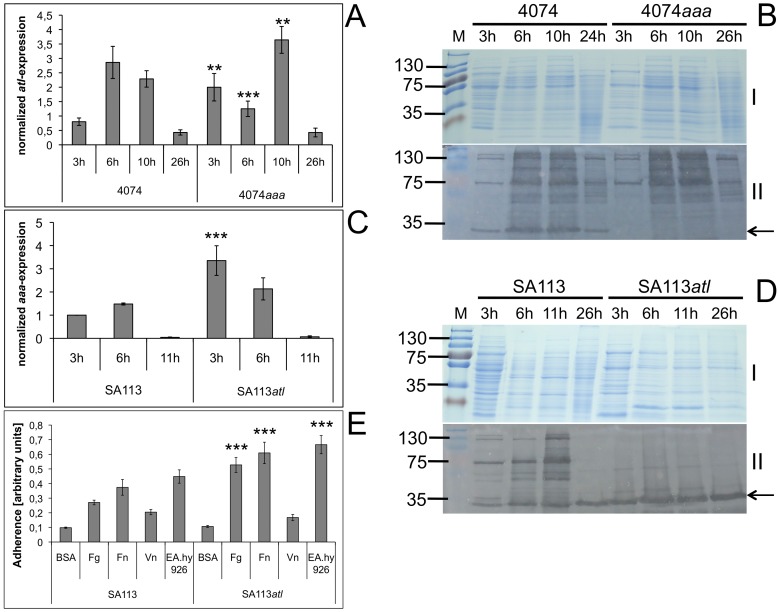
Expression analysis of *atl* and *aaa*. *atl* and *aaa* are upregulated in the 4074*aaa* and SA113*atl* mutant, respectively (A–D), and upregulation of *aaa* in the SA113*atl* mutant correlates with an increased adherence to Fg, Fn, and endothelial cells (E). The expression of the *atl* and *aaa* gene was determined by real-time PCR analysis and normalized to the expression of the housekeeping genes *gmk*, *aroE*, and *gyrB*. A: The level of *atl* mRNA in the 4074*aaa* mutant was significantly higher after 3 h and 10 h of growth and significantly lower after 6 h of growth compared to the 4074 wild type. Results are shown as the mean of three independent experiments. Statistical significance is indicated by asterisks. B: SDS-PAGE (10% separation gel, I) and semi-quantitative zymographic analysis (II) of surface-associated proteins from the 4074 wild type and the 4074*aaa* mutant showed increased lytic activity due to Atl (138 kDa) and its cleavage products producing a clearing zone ranging from ∼51 kDa to ∼130 kDa. The arrow indicates Aaa-associated bacteriolytic activity. C: The level of *aaa* mRNA was significantly and three-fold elevated in SA113*atl* compared to the wild type in the early exponential growth phase (3 h). Moreover, after 6 h and 11 h, the level of *aaa* mRNA was slightly higher in the SA113*atl* mutant, which however was not statistically significant. Results are shown as the mean of three independent experiments. Statistical significance is indicated by asterisks. D: SDS-PAGE (10% separation gel, I) and semi-quantitative zymographic analysis (II) of surface-associated proteins from the SA113 wild type and the SA113*atl* mutant revealed a more pronounced band with bacteriolytic activity at the molecular size of ∼34 kDa in the SA113*atl* mutant, reflecting an increased production of Aaa. The arrow indicates Aaa-associated bacteriolytic activity. E: The adhesive properties of the SA113 wild type and the SA113*atl* mutant were analyzed by ELISA adherence assays. The SA113*atl* mutant was significantly higher adherent to immobilized Fg, Fn, and EA.hy 926 cells than the SA113 wild type. In contrast, the Vn-binding activity of the SA113*atl* mutant did not significantly differ from the SA113 wild type. Results are shown as the mean of three independent experiments. Statistical significance is marked by asterisks.

To address this question, we analyzed the expression of *atl* in the 4074*aaa* mutant as well as the expression of *aaa* in the previously reported SA113*atl* mutant [21] by real-time PCR analysis and also studied the adhesive capacities of the SA113*atl* mutant. Indeed, real-time PCR analysis revealed a significantly higher level of *atl* mRNA in the 4074*aaa* mutant compared to the wild type after 3 h and 10 h of growth (Fig. 4A), reflecting an increased expression of *atl*. After 26 h of growth, there was no marked difference between the 4074*aaa* mutant and its wild type. Surprisingly after 6 h of growth, the level of *atl* mRNA was significantly lower in the 4074*aaa* mutant compared to the wild type. Semi-quantitative zymographic analysis of surface-associated proteins of 4074*aaa* cultures taken at different time points (Fig. 4B II) demonstrated increased zones of lytic activity with molecular sizes ranging from ∼52 kDa to ∼130 kDa in comparison with the wild type after 3 h, 6 h, and 10 h of growth (Fig. 4B II), which results from the action of Atl and its degradation products. A band with lytic activity due to Aaa is missing from the 4074*aaa* mutant (Fig. 4B II).

Furthermore, real-time PCR analysis revealed a statistically significant higher level of *aaa* mRNA in the SA113*atl* mutant (approximately three-fold) compared to its wild type in the early exponential growth phase (3 h) (Fig. 4C), reflecting an increased expression of *aaa*. Higher amounts of *aaa* mRNA were also detected in the SA113*atl* mutant after 6 h and slightly also after 11 h of growth, which however was not statistically significant. In agreement with our hypothesis and the results of real-time PCR analysis, semi-quantitative zymographic analysis of surface-associated proteins from cultures taken at different time points revealed stronger lytic bands at the molecular size of ∼34 kDa (corresponding to Aaa) for the SA113*atl* mutant compared to the wild type (Fig. 4D II). Bands with lytic activity due to Atl are missing from the SA113*atl* mutant (Fig. 4D II).

In order to investigate the result of the loss of Atl and the simultaneous increase in production of Aaa in the SA113*atl* mutant on *S. aureus* adherence to plasma and ECM proteins and human endothelial cells, we examined the adhesive properties of the SA113*atl* mutant by ELISA adherence assays. Interestingly, the SA113*atl* mutant revealed a significantly increased adherence to immobilized Fg, Fn, and endothelial cells in comparison to its wild type, but not to Vn (Fig. 4E). The increased adherence might be attributed to the increased expression of Aaa.

### Expression of *aaa* is Controlled by *agr* and SarA

**Figure 5:**
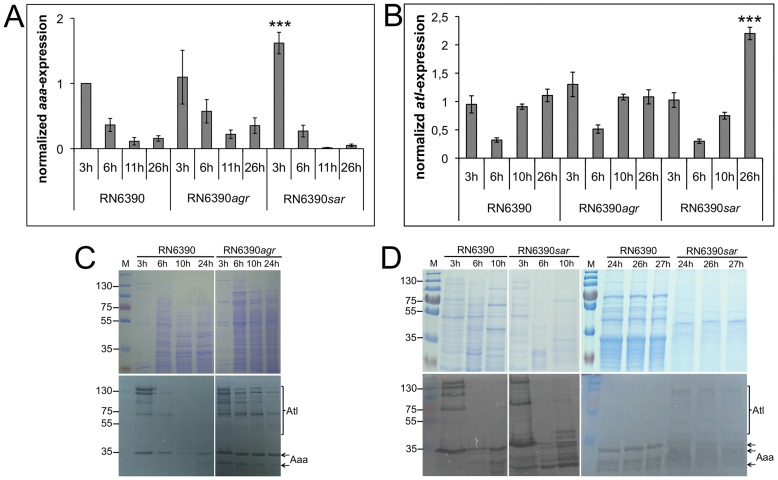
Impact of global virulence regulators on *aaa* expression. The expression of *aaa* (A) or *atl* (B) in the RN6390 wild type and its RN6390*agr* and RN6390*sarA* mutants was assessed by real-time PCR analysis. The results are shown as the mean of three independent experiments and statistical significance is marked by asterisks. A: The expression of *aaa* is significantly increased during the early exponential growth phase (3 h) in the RN6390*sarA* mutant compared to the wild type. An increased expression of *aaa* was also observed in the RN6390*agr* mutant after 6 h, 11 h, and 26 h of growth, which however was not statistically significant. B: The expression of *atl* in the RN6390*agr* mutant was higher after 3 h of growth compared to the wild type, which however did not reach statistical significance. In the RN6390*sarA* mutant, a significantly higher level of *atl* mRNA was detected after 26 h of growth. C, D: Aaa and Atl protein production was assessed by semi-quantitative SDS-PAGE (10% separation gel) (upper panel) and corresponding zymographic analysis (lower panel) of surface-associated proteins of the RN6390 wild type and the RN6390*agr* mutant (C) or the RN6390 wild type and the RN6390*sarA* mutant (D). Aaa and Atl protein production was much more pronounced with the RN6390*agr* mutant than with the RN6390 wild type after 6 h, 10 h, and 24 h of growth, strongly indicating a negative regulatory effect of *agr* on the expression of both, *aaa* and *atl*. Aaa protein production was more pronounced with the RN6390*sarA* mutant than with the RN6390 wild type after 3 h, 6 h, and 10 h of growth, indicating a negative regulatory effect of *sarA* on the expression of *aaa*.

Until now, nothing has been reported about the regulation of the expression of *aaa*. Therefore, we determined whether or not the expression of *aaa* depends on global staphylococcal virulence gene regulators, such as the accessory gene regulator *agr* and the staphylococcal accessory regulator *sarA.* For this, we determined the expression of *aaa* in the *S. aureus* strain RN6390 and its isogenic *agr* (RN6390*agr*) and *sarA* (RN6390*sarA*) mutant strains [31], [32] by real-time PCR analysis. In the RN6390*sarA* mutant, the level of *aaa* mRNA was significantly increased in the early exponential growth phase (3 h), indicating a negative regulatory impact of *sarA* on the expression of *aaa* (Fig. 5A). In the RN6390*agr* mutant, the level of *aaa* mRNA was increased after 6 h, 11 h, and 26 h of growth compared to the RN6390 wild type. However, these differences did not reach statistical significance (Fig. 5A). To further verify whether the expression of *aaa* is under the control of *agr* and *sarA*, we assessed the level of Aaa protein production in the RN6390 wild type and the RN6390*agr* and RN6390*sarA* mutants by semi-quantitative SDS-PAGE and corresponding zymographic analysis of surface-associated proteins from RN6390, RN6390*agr*, and RN6390*sarA* cultures taken at different time points. The RN6390 wild type produced a much lower level of Aaa after 6 h, 10 h, and 24 h of growth compared to the RN6390*agr* mutant and also after 3 h, 6 h, and 10 h of growth compared to the RN6390*sarA* mutant, strongly supporting an *agr*- and *sarA*-dependent expression of *aaa* (Fig. 5C and D; lower panel). In the RN6390*sarA* mutant, the level of the Aaa degradation products was strongly increased (Fig. 5D; lower panel), which may be explained by a strongly increased production of extracellular proteases that was observed with the *sarA* mutant [33].

Furthermore, we determined the expression of *atl* in the RN6390 wild type and the RN6390*agr* and RN6390*sarA* mutant strains using real-time PCR analysis. In the RN6390*sarA* mutant, we observed a significantly increased expression of *atl* after 26 h of growth in comparison with the RN6390 wild type (Fig. 5B). In the RN6390*agr* mutant, the level of *atl* mRNA was slightly higher after 3 h, 6 h, and 10 h of growth than in the RN6390 wild type, which however (as with *aaa*) was not statistically significant (Fig. 5B). As in the case of Aaa, semiquantitative zymographic analysis revealed a much lower level of Atl production by the RN6390 wild type compared with the RN6390*agr* mutant after 6 h, 10 h, and 24 h of growth, indicating an *agr*-dependent expression of *atl* (Fig. 5C; lower panel). In agreement with the data from real-time PCR analysis, semi-quantitative zymographic analysis revealed that the Atl-associated bacteriolytic activity was not much different between the RN6390*sarA* mutant and its wild type after 3 h, 6 h, and 10 h of growth (Fig. 5D; lower panel). Furthermore, in agreement with an increased *atl* mRNA expression in the RN6390*sarA* mutant after 26 h of growth, the level of Atl-associated bacteriolytic activity was elevated in the RN6390*sarA* mutant after 24 h, 26 h, and 27 h of growth (Fig. 5D; lower panel). As with Aaa, Atl also seemed to be partially degraded very likely due to the increased production of proteases by the *sarA* mutant [33].

## Discussion

The adherence of *S. aureus* to host factor-coated material or host tissue is an important step in the initiation of *S. aureus* infections. In this study, we analyzed the functional modular structure of Aaa. Interestingly, SPR revealed that both the LysM domain and the CHAP domain of Aaa mediate binding to Fg, Fn, and Vn dose-dependently and with high affinity. The *K*_D_ value calculated for the binding of Aaa to Vn was very similar to that reported previously (18 nM versus 22.1 nM) [22]. The respective *K*_D_ values for the binding of Aaa to Fn and Fg were 5 to 15 times lower than those observed previously (6.0 nM versus 29.9 nM for Fn and 0.76 nM versus 12.3 nM for Fg) [22], indicating even stronger binding. The difference in results might be due to better protein qualities in the more recent study. The calculated *K*_D_ values were found to be very similar among Aaa, N-Aaa and C-Aaa. The stoichiometry of the binding of Aaa, N-Aaa, and C-Aaa to Fg is suggested to be 4∶2∶1 (Fig. 2 II A). In contrast, the binding of Aaa, N-Aaa, and C-Aaa to Fn and Vn appears to be 2∶1∶1 (Fig. 2 II B and C). This strongly indicates that multivalent interactions are involved in the binding of Aaa to these plasma and ECM proteins. Such multivalent interactions have also been described for other adhesins, such as the Fn-binding autolysin/adhesin AtlC from *S. caprae* [19] and FnBPA or FnBPB, where different and partially repeated domains of the proteins are involved in binding to Fg, elastin, and Fn [8].

This is the first study that has identified an adhesive function for a CHAP domain, i.e. binding to Fg, Fn, and Vn. Until now, only enzymatic (bacteriolytic) activities have been associated with CHAP domains. The 110-140 aa CHAP domain was independently described by two research groups and has been demonstrated to specifically cleave various amide bonds of peptidoglycan [34], [35]. Recently, a total of 163 CHAP domain-containing proteins from 12 staphylococcal genomes have been analyzed systematically. These proteins may be classified into two groups. The first group, bacterial CHAP proteins (CHAP enzymes) are encoded on a plasmid or the chromosome, as in the case of Aaa. The second group, phage CHAP proteins (CHAP lysins) are encoded by a phage/prophage [26]. The bacterial CHAP domains are all located within the proteins C-terminal portion, while the phage CHAP domains usually are located within the proteins N-terminal portion [26]. Staphylococcal CHAP domains can work with or without one of two common cell wall-targeting domains, LysM and SH3_5. While the bacterial CHAP proteins use LysM domains as their cell wall-targeting domain, phage CHAP proteins use SH3_5 domains [26]. Thus, Aaa represents a typical bacterial CHAP protein as it contains three LysM domains in its N-terminal portion (Fig. 1A). In the case of the well-described *N*-acetylglucosaminidase AcmA from *Lactococcus lactis*, the presence of three C-terminal LysMs appears to be essential for optimal lactococcal cell wall degradation [24]. We found that the Aaa CHAP domain has bacteriolytic acitivity without the need for the presence of the LysM domain. However, the LysM domain seems to be required for optimal activity.

The sequence similarities of 4 staphylococcal CHAP domains analyzed are surprisingly low (21% to 37% identity and 33% to 51% similarity in a pairwise comparison) [26], which could be the reason for potential additional activities associated besides the enzymatic activities, such as adhesive functions. Thus, it may be speculated that several multifunctional CHAP proteins harbor a variety of so far unidentified additional properties.

The LysM domain has been found in more than 4,000 proteins of prokaryotic or eukaryotic origin. It is typically 44 to 65 aa in length. In the case of prokaryotic proteins the LysM domain binds to various types of peptidoglycan thereby mediating non-covalent surface association of the protein [24]. To our knowledge, until now, the involvement of a LysM domain in adherence to host factors, has not been described yet. We found that the LysM domain binds to Fg, Fn, and Vn. Most likely, the LysM domain recognizes the *N*-acetylglucosamine moiety of peptidoglycans [24]. Thus, it may be speculated that LysM-mediated binding of Aaa to plasma and ECM proteins occurs via the carbohydrate moiety of these glycoproteins.

LysM domains also occur in other *S. aureus* adhesins: The immunoglobulin- and von Willebrand factor-binding protein A as well as the elastin-binding protein EbpS each carry one copy of the LysM domain [36], [37]. In these cases, the LysM domain may not be responsible for the surface attachment, but it may be required for proper positioning of the protein, because protein A is covalently bound to the peptidoglycan via an LPXTG-motif and EbpS is an integral membrane protein [24]. Noteworthy, the LysM domain does not seem to be involved in the adhesive functions of protein A and EbpS.

Although the purified recombinant Aaa binds to ECM proteins, we did not detect decreased adherence of the 4074*aaa* mutant or increased adherence of the complemented 4074*aaa* mutant to Fg or Fn, but only to Vn. A possible explanation of why the 4074*aaa* mutant did not show markedly reduced binding to the ECM proteins Fg and Fn compared to its wild type might be that *S. aureus* has been known to express a multitude of adhesins with redundant functions: examples include the Fn- and Fg-binding MSCRAMMs (FnBPs and clumping factors ClfA and ClfB) [38], the SERAMs (secretable expanded repertoire adhesive molecules) [39], such as the Fg- and Fn-binding proteins Eap and Emp [39], and the autolysin/adhesins [20]. The FnBPs mediate binding to Fn, Fg, and elastin and the clumping factors mediate binding to Fg, cytokeratin 10, and epithelial cells (reviewed in [8]). Thus, if one factor is missing, its function might be taken over by many other adhesins. Indeed, mutants deficient in *fnbA* or *fnbB* do not reveal significantly reduced binding to Fn [40]. Furthermore, we did not detect a reduced internalization rate of the 4074*aaa* mutant by endothelial cells. This indicates that, unlike Atl, Aaa does not play a role in internalization. Aaa lacks the glycine-tryptophan (GW)-dipeptide motifs, which are most likely responsible for the internalization mediated by Atl [20], but a possible involvement of Aaa in internalization was assumed, since a similar function has been described for the LysM domain-containing protein p60 [28]. However, because of potentially masking effects by the FnBPs and Atl, a low level involvement of Aaa in internalization cannot be completely ruled out at this time.

Alternatively or additionally, the partial lack of adherence defects in the 4074*aaa* mutant might also be explained by the increased expression of the autolysin/adhesin Atl in the 4074*aaa* mutant that we observed in comparison with its wild type. Indeed, the results of the present study strongly suggest that one autolysin/adhesin can compensate for the loss of the other and that redundant Aaa- and Atl-mediated functions are at least partially interchangeable: The SA113*atl* mutant that showed enhanced adherence to Fg, Fn, and endothelial cells also exhibited increased expression of *aaa* and production of Aaa as determined by real-time PCR and zymographic analysis.

The expression of *S. aureus* virulence factors is under the control of global virulence gene regulators, such as the two-component signal transduction system *agr* or the DNA-binding transcription factor SarA [31]. Until this time, nothing has been reported about the regulation of *aaa*. Therefore, we determined the abundance of *aaa* mRNA and *atl* mRNA in *S. aureus* RN6390, which lacks the global virulence regulators *agr* and SarA, by real-time PCR analysis. We detected a negative regulatory impact of both, *agr* and SarA, on the expression of *atl* thereby confirming previous findings: In an *agr* mutant as well as in a *sarA* mutant of *S. aureus* strain UAMS-1, a significantly higher Atl protein abundance was detected compared to the wild type [41]. Moreover, our results indicated a negative regulatory effect of both, *agr* and SarA, on the expression of *aaa*. Thus, in this respect, the expression of *aaa* seems to be controlled in a similar way like *atl*. Additionally, further and so far unknown regulatory mechanisms seem to be involved, because the bacteria might sense the level of one autolysin (Aaa or Atl) and increase the level of the other (Atl or Aaa), if the first one is lacking.

In conclusion, Aaa represents a virulence factor probably by promoting *S. aureus* attachment to host factor-coated material and host tissue. In this study, we have demonstrated that both the Aaa LysM domain and the CHAP domain can mediate binding to the plasma and ECM proteins Fg, Fn, and Vn, thus representing novel functions for these widely distributed protein domains.

## Materials and Methods

### Bacterial Strains, Growth Conditions, Plasmids, and Cell Culture

Bacterial strains used in this study are listed in Table 1. *Staphylococcus* and *Escherichia coli* strains were grown aerobically at 37°C in Tryptic Soy (TS) broth (TSB, BD Bioscience) and Luria Bertani (LB) medium (BD Bioscience), respectively. TS and LB agar plates contained 1.4% agar. The production of 6 x Histidine (His)-tagged fusion proteins in *E. coli* was induced by supplementing the culture with 1 mM IPTG. Antibiotics were added, when appropriate: Chloramphenicol (Cm; 10 µg/ml; Roth, Karlsruhe, Germany), erythromycin (Em; 10 µg/ml; Merck, Darmstadt, Germany), spectinomycin (Spc; 150 µg/ml; Sigma Aldrich, München, Germany), tetracycline (Tc; 10 µg/ml; Applichem, Darmstadt, Germany), and ampicillin (Am; 100 µg/ml; Serva, Heidelberg, Germany). For the production and purification of the 6 x His-fusion proteins (Aaa, N-Aaa, C-Aaa), the vector pQE30 (Qiagen, Hilden, Germany) and for the complementation of the 4074*aaa* mutant, the shuttle vector pRB473 [42] was used. For the internalization and adherence assays, the endothelial cell line EA.hy 926 was employed [43]. Cultivation of the EA.hy 926 cells was performed as described recently [20].

**Table 1:** Bacterial strains used in this study.

Strain	Relevant genotype/plasmid	Relevant properties	Source/reference
***S. aureus***			
Cowan 1	reference isolate	isolated from septic arthritis	ATCC 12598; NCTC 8530
4074	clinical isolate	isolated from endocarditis	[48]
4074*aaa*	4074 *aaa*::*ermB*; Em^r^	deficient in autolysin Aaa	[22]
4074*aaa* (pRCK*aaa*)	4074 *aaa*::*ermB*; Em^r^ (pRCK*aaa*); Cm^r^	complemented with *aaa*	this study
SA113	ATCC 35556; NCTC 8325 derivative	accepts foreign DNA	[49]
SA113*atl*	SA113 *atl*::*spc*; Spc^r^	deficient in autolysin Atl	[21]
RN6390	NCTC 8325 derivative, *rsbU* ^–^	reference isolate	[31]
RN6390*agr*	RN6390 *agr::tet*; Tc^r^	deficient in accessory gene regulator *agr*	[31]
RN6390*sarA*	RN6390 *sarA::ermB*; Em^r^	deficient in staphylococcal accessoryregulator *sarA*	[50]
***S. carnosus***			
TM300	reference isolate	non pathogenic	[51]
***E. coli***			
TG1	*supE hsd*Δ*5 thi*Δ*(lac-proAB) F’(traD36* *proAB^+^ lacI^q^ lacZ*Δ*M15)*	cloning and expression host	[52]
	TG1 (pQA35); Am^r^	producing Aaa	[22]
	TG1 (pQaC4074); Am^r^	producing C-Aaa	this study
	TG1 (pQaN4074); Am^r^	producing N-Aaa	this study

### DNA Manipulations, Transformation, Polymerase Chain Reaction (PCR), DNA Sequencing, Websites, and Accession Numbers

DNA manipulations and transformation of *E. coli* were performed according to standard procedures [44]. *S. aureus* 4074*aaa* was transformed by protoplast transformation [45]. Plasmid DNA was isolated using the QIAprep Spin Miniprep Kit (Qiagen) and staphylococcal genomic DNA was isolated with the QIAamp DNA Blood Mini Kit (Qiagen). PCR was carried out using the PCR Extender System (5 Prime; distributed by VWR International) according to the instructions of the manufacturers. The primers (Table 2) were synthesized by Eurofins MWG Operon (Ebersberg, Germany). DNA sequences were determined by Eurofins MWG Operon using their standard sequence primers pQEfor and pQErev and others (Table 2) and an ABI 3730XL DNA sequencer. The DNA and deduced aa sequences were analyzed using the program JustBio at http://www.justbio.com.

**Table 2:** Primers used in this study.

Primer name	Primer sequence (restriction site underlined)
CH58^1^	5′-GCACTGCAGCGTAAGACTTTAGTGAATATATC-3′
CH59^1^	5′-CAGGGATCCCATGCTTATGTTTGTAGGGCG-3′
C-Aaa-F^1,2^	5′-CAGGGATCCACAAATAGAGGTTACAATACACCAG-3′
C-Aaa-R^1,2^	5′-GCACTGCAGCATTATATATTTATATACGTAAGAC-3′
N-Aaa-F^1,2^	5′-CAGGGATCCGCTACAACTCACACAGTAAAACCGGG-3′
N-Aaa-R^1,2^	5′-GTCCTGCAGTTACGTTGTTGCAGATCCTGAGTTCGTAG-3′
*aroE*-F^3^	5′-CTATCCACTTGCCATCTTTTAT-3′
*aroE*-R^3^	5′-ATGGCTTTAATATCACAATTCC-3′
*gmk*-F^3^	5′-AAGGTGCAAAGCAAGTTAGAA-3′
*gmk*-R^3^	5′-CTTTACGCGCTTCGTTAATAC-3′
*gyrB*-F^3^	5′-AATTGAAGCAGGCTATGTGT-3′
*gyrB*-R^3^	5′-ATAGACCATTTTGGTGTTGG-3′,
*aaa*-F^3^	5′-TACGAACTCAGGATCTGCAA-3′
*aaa*-R^3^	5′-GCTGCGTTATCCCAGTTATT-3′
*atl*-F^3^	5′-ATGGATACGAAGCGTTTAGC-3′
*atl*-R^3^	5′-CACTACATCTGCACCTTTCG-3′
pRBseq-1^2^	5′-AGATCCAGTAATGACCTCAG-3′
pRBseq-2^2^	5′-TACCCCAGGCGTTTAAGG-3′

Primers used for ^1^cloning, ^2^sequencing, ^3^real-time PCR analysis.

### RNA Preparation, cDNA Synthesis, and Real-time PCR

Total RNA from *S. aureus* was isolated using the RNeasy Mini Kit (Qiagen). TSB medium was inoculated with staphylococci to an OD_578_ of 0.05 and cultivated aerobically at 37°C. 4 ml samples were taken at different time points and immediately mixed with an equal volume of RNAprotect Bacteria Reagent (Qiagen). After 5 min of incubation at ambient temperature, cells were harvested (10 min, 4,000×*g*, 4°C), resuspended in 1 ml RNA lysis tissue (RLT) buffer (Qiagen), supplemented with 10 µl ß-mercaptoethanol (Applichem) and mechanically disrupted using Lyzing Matrix B silica spheres (MP Biomedicals) and the FastPrep®-24 instrument (MP Biomedicals). Further RNA purification steps were performed according to the protocol of the manufacturer. RNA quantity and quality was determined using the RNA 6000 Nano Kit and the 2100 Bioanalyzer (Agilent Technologies) of the Integrated Functional Genomik IFG, Münster, Germany.

cDNA was synthesized using 1 µg of template RNA and the QuantiTect Reverse Transcription Kit (Qiagen) according to the manufacturer. After the initial removal of genomic DNA, small samples were taken from each vial for validation of complete genomic DNA removal during real-time data collection. Real-time PCR was performed on an iQ^TM^5 real-time PCR Detection System (Biorad) using the iQ™ SYBR® Green Supermix (Biorad) and 400 nM of the primer pairs *aroE*-F/*aroE*-R, *gmk*-F/*gmk*-R, *gyrB*-F/*gyrB*-R, *aaa*-F/*aaa*-R, and *atl*-F/*atl*-R (Table 2). The PCR conditions were: 1 cycle at 95°C for 3 min, followed by 35 cycles of 10 s at 95°C, 30 s at 55°C and 30 s at 72°C. The PCR amplification was performed in duplicate. Melting curve analysis ensured PCR specificity. The expression of *aaa* or *atl* in *S. aureus* SA113 after 3 h of growth was defined as an internal control. Gene expression levels of *aaa* or *atl* were normalized to the expression of the housekeeping genes *gmk* (encoding guanylate monophosphate kinase), *aroE* (encoding shikimate dehydrogenase) and *gyrB* (encoding the subunit B of DNA gyrase) by means of the iQ^TM^5 Optical System Software Version 2.0 (Biorad).

### Complementation of *S. aureus* 4074*aaa*

The *aaa* gene was amplified by PCR from *S. aureus* 4074 genomic DNA using the primers CH58 and CH59 (Table 2) yielding a 1,324 bp DNA fragment. The DNA fragment was cloned into the PstI and BamHI sites of the shuttle vector pRB473 in *E. coli*, generating the plasmid pRCK*aaa*. The insert of the plasmid pRCK*aaa* was verified by DNA sequencing and pRCK*aaa* was introduced into *S. aureus* 4074*aaa*.

### Expression and Purification of 6 x His-N-Aaa and 6 x His-C-Aaa Fusion Proteins from *E. coli*

The expression and purification of the whole recombinant 6 x His-Aaa (Aaa: ∼34 kDa) was decribed earlier [22]. To characterize the modular structure of Aaa, His-tagged Aaa subdomains representing the N-terminal LysM domain (N-Aaa: ∼22 kDa) or the C-terminal CHAP domain (C-Aaa: ∼13 kDa) were constructed. For this, DNA sequences encoding N-Aaa or C-Aaa were PCR-amplified from *S. aureus* 4074 genomic DNA using the primers N-Aaa-F and N-Aaa-R or C-Aaa-F and C-Aaa-R (Table 2). The resulting DNA fragments (N-*aaa*: 570 bp; C-*aaa*: 360 bp) were cloned into the BamHI and PstI sites of the vector pQE30 in *E. coli* so that the respective gene products are in frame with the codons for 6 x His creating the plasmids pQaN4074 or pQaC4074. Correct clones were verified by DNA sequencing. Purification of the 6 x His-fusion proteins from *E. coli* cultures containing plasmid pQaN4074 or pQaC4074 was performed under denaturing conditions using Ni-NTA superflow columns (Qiagen) according to the protocol of the manufacturer.

### Protein Isolation, SDS-PAGE, and Zymographic Analysis

Surface-associated proteins of staphylococcal strains were prepared essentially as decribed before [22]. Briefly, bacterial cells were grown for the times indicated, then 20 ml of the cultures were harvested by centrifugation. Resulting cell pellets were resuspended in 1 volume of 4 x Laemmli (SDS) sample buffer, heated for 5 min at 95°C, and centrifuged again.

Purified Aaa, N-Aaa, C-Aaa (2 µg), or surface-associated proteins were separated by SDS-PAGE (15% or 10% separation gel, 4.5% stacking gel) and stained with Coomassie brilliant blue G250. Zymographic analysis of the bacteriolytic activities of protein preparations was performed essentially as described before [20]. Staphylococcal surface-associated proteins or purifed proteins were separated by SDS-PAGE on a polyacrylamide gel containing heat-inactivated *S. carnosus* cells (0.2%) as a substrate for lytic enzymes in the separation gel. For semi-quantitative zymographic analysis to determine Aaa and Atl protein production, 10 µl of surface-associated proteins of *S. aureus* strains were separated by SDS-PAGE (10% separation gel) and on a corresponding zymogram. After electrophoresis, the gels were washed and incubated overnight in 25 mM Tris-HCl buffer (pH 8.0) containing 1% (v/v) Triton X-100 (Sigma Aldrich) at 37°C. Bands with bacteriolytic activity were observed as clear zones in the opaque gel. After photography against a dark background, the clear zones appeared as dark bands. Sometimes, the gels were stained with 1% (w/v) methylene blue (Merck) in 0.01% (w/v) KOH after incubation in Tris-HCl buffer and destained with ddH_2_O. In these gels, bands with lytic activity were observed as clear zones in the blue stained gels.

### ELISA Adherence Assay

The wells of 96-well microplates were coated with the human proteins Fg (20 µg/ml; Calbiochem), Fn (10 µg/ml; Roche), Vn (5 µg/ml), or as a negative control with 1% BSA (Fraction V, Sigma Aldrich) at 4°C and subsequently blocked. Vn was purified from human serum according to Hayashi before [46]. To assess the adherence to endothelial cells, EA.hy 926 cells were grown to confluency in 96-well cell culture plates (Greiner Bio-One), washed with PBS, fixed with ice-cold methanol (Merck) and blocked with 1% BSA. Then, the microplates were washed thrice and each well was incubated for 1 h at 37°C with 100 µl of a staphylococcal suspension, which was previously grown overnight, washed with PBS, sonicated using an ultrasonic cell disruptor (Branson Sonifier® 250) to separate cell aggregates, and adjusted to an OD_578_ of 1.0 (corresponding to approximately 5×10^8^ cfu/ml). Special emphasis was put on sonication to disrupt all cell clusters to exclude the possibility that the increased adherence of the SA113*atl* mutant was due to the defect in cell separation resulting in cell clumping and thus could represent a false positive result. Appropriate mechanical cell separation was verified by phase contrast light microscopy (not shown). Unbound bacterial cells were removed by washing thrice with 200 µl PBS. Bound *S. aureus* cells were detected by a polyclonal rabbit anti-*S. aureus* antibody (2 µg/ml; Abcam) and AP-conjugated goat anti-rabbit IgG (0.3 µg/ml; Dako). SigmaFast™ *p*-Nitrophenylphosphate (Sigma Aldrich) conversion was detected by determination of the optical density (OD_405_) after 30 min of incubation.

### Real-time Interaction Analysis Using SPR for Quantification of Molecular Interactions

Experiments were performed on a BIAcore®2000 instrument according to the general procedures recommended by the manufacturer of the instrument essentially as described previously [22]. Aaa, N-Aaa, and C-Aaa were immobilized on the sensor chip C1 to analyze their interactions with the ECM proteins Fg, Fn, and Vn. For immobilization, Aaa, N-Aaa, and C-Aaa were covalently coupled to the sensor chip surface C1 via primary amine groups. Low densities of the immobilized proteins on the chips were chosen to minimize avidity effects and mass transfer limitations. To prepare the surface of the sensor chip, a freshly prepared 50 mM NaOH solution at a flow rate of 50 µl/min was injected (3×50 µl). Afterwards, to activate the sensor chip, a mixture of 100 µl NHS/EDC (0.1 M N-hydroxysuccinimide [NHS], 0.4 M N-ethyl-Ǹ-(3-dimethylaminopropyl)-carbodiimide [EDC]; amine coupling kit, GE Healthcare) was injected at a flow rate of 10 µl/min. Aaa (approximately 2 µg/ml), N-Aaa (approximately 4 µg/ml), and C-Aaa (approximately 2 µg/ml) in 10 mM sodium acetate buffer (pH 5.0) were immobilized on the surface and remaining NHS-ester groups on the sensor chip surface were then blocked by injection of 100 µl 1 M ethanolamine (pH 8.5) (amine coupling kit, GE Healthcare). After Aaa was immobilized on the C1 sensor chip surface (149 pg Aaa/mm^2^; 96 pg N-Aaa/mm^2^; 72 pg C-Aaa/mm^2^), these different immobilization levels correspond to nearly equimolar surface densities of the three proteins according to their molecular masses), ECM proteins in increasing concentrations were injected over the sensor surface for the kinetic studies at a flow rate of 50 µl/min. Binding of ECM proteins was monitored and presented in an overlay-plot of the sensorgrams (a plot of RU [resonance unit] versus time). Kinetic data were determined at 25°C. For regeneration of the sensor chip surface after the injection of Fg and Fn, 100 mM glycine, pH 10.5 (3x 1 min) was used resulting in a return to baseline. For regeneration of the sensor chip surface after the injection of Vn, 100 mM glycine, pH 12/0.3% triton (3x 1 min) followed by 100 mM glycine, pH 10.5 (3x 1 min) was used resulting in a return to baseline. Kinetic data were analyzed using BIA-evaluation software (version 4.1) from Biacore AB as described [47].

### Preparation of FITC-labeled Staphylococci and Plasma and ECM Proteins

Overnight-grown staphylococci were washed, sonicated, fixed, and fluorescein isothiocyanate (FITC isomer I; Invitrogen, Karlsruhe, Germany)-labeled as described before [20]. Like with the ELISA adherence assay (see above) and the internalization assay described before [20], we put special emphasis on sonication to disrupt all cell clusters to ensure that the increased adherence of the SA113*atl* was not due to the pronounced cell clumps formed by the mutant. 1 mg/ml of Fg, Fn, Vn, or BSA were labeled with 800 µg/ml FITC in Ca^2+^-TBS (150 mM NaCl, 25 mM Tris, 2 mM KCl, 2 mM CaCl_2_, pH 7.4) for 2 h at ambient temperature under gentle agitation and protected from light, followed by overnight incubation at 4°C. Insoluble FITC was removed by centrifugation (15 min, 17,000×*g*). Excessive, soluble FITC was separated from FITC-coupled proteins using Amicon® Ultra Centrifugal Filters Ultracel® –10K (Millipore) and Ca^2+^-TBS. The labeled protein solutions were washed 6 times including in-between centrifugation (15 min, 4,000×*g*) until the washing solution remained colorless. The final concentration of labeled proteins was determined using the BCA Protein Assay (Thermo Scientific).

### Flow-cytometric Adherence and Internalization Assays

Adherence of FITC-labeled plasma and ECM proteins to staphylococci was assessed by flow cytometry. Overnight-cultured staphylococci were washed with Ca^2+^-TBS (see above) and adjusted to an OD_578 nm_ of 1.0 in Ca^2+^-TBS. 10 µl of the respective bacterial solutions (5×10^6^ cfu) were incubated with 5 to 200 µg/ml FITC-labeled Fg, Fn, Vn, or as a negative control with BSA for 30 min at ambient temperature and protected from light in a total volume of 50 µl. All samples were washed with 500 µl Ca^2+^-TBS, finally resuspended in 400 µl Ca^2+^-TBS, and gently sonicated in a water bath. The main population of intact and separated bacteria was gated in a two-parameter dot plot referring to the sideward scatter (SSC) and the forward scatter (FSC) of unlabeled bacteria on a FacsCALIBUR™ (BD Bioscience) using the CellQuest™ pro acquisition and analysis software (BD Bioscience). To exclude acquired bacterial autofluorescence from data analyses, we adjusted the median fluorescence signal of unlabeled bacteria to 20–30 channel volumes. FITC-labeled proteins bound to the staphylococci were detected in the fluorescence channel 1 (FL-1, 530/30 bandpass filter) in a one-parameter histogram, analyzing 5,000 cells per sample. For statistical analyses, median fluorescence values were used. Sample preparation and detection of internalized staphylococci by EA.hy 926 cells were performed by flow-cytometric internalization assays as described before [20].

### Statistical Analysis

Mean values of experimental data were compared with one-way ANOVA and, if adequate with subsequent Bonferroni’s posttest for multiple comparisons using GraphPad InStat3. *P* values ≤0.05 were considered statistically significant and are indicated with asterisks: * (*P*≤0.05), ** (*P*≤0.01), and *** (*P*≤0.001).
